# Supplementary material for: Preferences, trust, and performance in youth business groups
Source: PLoS One. 2021 Sep 20;16(9):e0257637. doi: 10.1371/journal.pone.0257637 (PMC8452030; doi:10.1371/journal.pone.0257637)
Supplement: S1 Appendix — (DOCX) [file pone.0257637.s001.docx]

# Supporting information

## S1 Appendix. Experimental protocols

| **Game set 1. To elicit social preferences** | | | |
| --- | --- | --- | --- |
| a. We will introduce to you **eight** sharing games where you will decide what you prefer.  b. You will have a chance to earn money by participating in these games and your **answers** will affect how much you and some others will get.  c. Only one game will result in payout but you do not know which one when you make your **answers**.  d. A lottery will determine which ones will be for real after all the games are played.  e. By making careful **answers** in each game, you have a greater chance of getting your preferred payout. | | | |
| S1 | **Sharing game 1**: You can choose between two sharing options **between yourself and another unknown** **member of your own youth group:**  **Option 1: 20 ETB** for yourself AND **20 ETB** for another  **unknown** member of your own youth group  **Option 2: 20 ETB** for yourself AND **0 ETB** for another  **unknown** member of your own youth group | Choice of sharing option: 1 or 2 |  |
| S2 | **Sharing game 2**: You can choose between two sharing options **between yourself and an unknown member of another youth group in your woreda:**  **Option 1: 20 ETB** for yourself AND **20 ETB** for another **unknown** member of another youth group in the woreda  **Option 2**: **20 ETB** for yourself AND **0 ETB** for another **unknown** member of another youth group in the woreda | Choice of sharing option: 1 or 2 |  |
| S3 | **Sharing game 3**: You can choose between two sharing options **between yourself and another unknown** **member of your own youth group:**  **Option 1: 20 ETB** for yourself AND **20 ETB** for another **unknown** member of your own youth group  **Option 2: 20 ETB** for yourself AND **40 ETB** for another **unknown** member of your own youth group | Choice of sharing option: 1 or 2 |  |
| S4 | **Sharing game 4**: You can choose between two sharing options  **between yourself and an unknown member of another youth group in your woreda:**  **Option 1: 20 ETB** for yourself AND **20 ETB** for another **unknown** member of another youth group in the woreda  **Option 2: 20 ETB** for yourself AND **40 ETB** for another **unknown** member of another youth group in the woreda | Choice of sharing option: 1 or 2 |  |
| S5 | **Sharing game 5**: You can choose between two sharing options **between yourself and another unknown** **member of your own youth group:**  **Option 1**: **20 ETB** for yourself AND **20 ETB** for another **unknown** member of your own youth group  **Option 2: 40 ETB** for yourself AND **0 ETB** for another **unknown** member of your own youth group | Choice of sharing option: 1 or 2 |  |
| S6 | **Sharing game 6:** You can choose between two sharing options **between yourself and an unknown member of another youth group in your woreda**:  **Option 1: 20 ETB** for yourself AND **20 ETB** for another  **unknown** member of another youth group in the woreda  **Option 2: 40 ETB** for yourself AND **0 ETB** for another  **unknown** member of another youth group in the woreda | Choice of sharing option: 1 or 2 |  |
| S7 | **Sharing game 7**: You can choose between two sharing options **between yourself and another unknown** **member of your own youth group:**  **Option 1**: **20 ETB** for yourself AND **20 ETB** for another  **unknown** member of another youth group in the woreda  **Option 2: 30 ETB** for yourself AND **40 ETB** for another  **unknown** member of another youth group in the woreda | Choice of sharing option: 1 or 2 |  |
| S8 | **Sharing game 8**: You can choose between two sharing options **between yourself and another unknown** **member of another youth group in your woreda**:  **Option 1**: **20 ETB** for yourself AND **20 ETB** for another  **unknown** member of another youth group in the woreda  **Option 2: 30 ETB** for yourself AND **40 ETB** for another  **unknown** member of another youth group in the woreda | Choice of sharing option: 1 or 2 |  |
| G1(S1-S8) | **Lottery to determine which of the games is real will take place at the end of the survey interview** |  |  |

| **8. Trust Game. General Instructions**  **You will now play two games related to trust, one will be with another anonymous member of your own group and one with another anonymous group member of another youth group in your own woreda. You will never find out who these members are and they will not find out who you are. One of these games will afterward be randomly selected /by throwing the die) as a real game that will be implemented. We are responsible for the transfer of the money between you and that other person by use of envelopes.**  **8.a.: Trust Game Within Own Youth Group (same format for 8.b.: With Youth Member in another Youth Group in the Woreda).**  This is an experiment where you will decide how much you trust other persons within your own youth group (by giving them money they are free to return some of to you). The anonymous person in your group that you are free to decide to invest in is free to return some, all or nothing to you of the amount you invest and we triple. Like you, that other person only knows that you are an anonymous member of your own youth group. The maximum tripled amount that can be invested by you in that other anonymous person is 90 ETB. Alternatively, you may keep 30 ETB for yourself if you do not trust that other person to return any of the amount given to that anonymous person in your own group. We ask you a sequence of questions in the form of tow alternatives that you have to choose between in each case. This is to identify how much you are willing to invest in that anonymous person in your own group and that indicates how much you trust that person based on how much you expect that person voluntarily will return to you. You will respond to a number of paired alternatives where you are free to choose the one you prefer for each of the two alternatives.  After you have completed this game for a member of your own group you will do the same for an anonymous group member of another youth group in your own woreda. | | | | |
| --- | --- | --- | --- | --- |
| 8a.1 | Do you agree to play the game? 1=Yes, 2=No | Code | |  |
| 8a.2 | What do you prefer of these two alternatives?   1. Invest 30 ETB in the trust game with another anonymous person in your own youth group. This amount will be tripled such that that person gets 90 ETB and is free to return some, all or nothing of that amount to you, or 2. Keep the whole 30 ETB for yourself and invest nothing in the anonymous member in your own group as you do not trust this person.   If choice 1, go to **8a.3**. If choice 2, go **8a.4.** | Code | |  |
| 8a.3 | What do you prefer?   1. Invest 30 ETB in the trust game with another anonymous person in your own youth group. This amount will be tripled such that that person gets 90 ETB and is free to return some, all or nothing of that amount to you, or 2. Keep 15 ETB for yourself AND invest 15 ETB in the trust of the anonymous member in your own group, which will be tripled to 45 ETB and who is free to return some, all or nothing to you.   If choice 1, go to **8a.4**. If choice 2, go **8.a.6** | Code | |  |
| 8.a.4 | What do you prefer?   1. Keep the whole 30 ETB for yourself AND invest nothing in the anonymous member in your own group as you do not trust this person, or 2. Keep 25 ETB for yourself AND invest 5 ETB in the trust of the anonymous member of your own group, which we triple to 15 ETB and the anonymous person is free to return some, nothing or all of that 15 ETB to you.   If choice 1, go to **Next experiment**. If choice 2, go to **8.a.9** | Code | |  |
| 8.a.5 | What do you prefer?   1. Invest 30 ETB in the trust game with another anonymous person in your own youth group. This amount will be tripled such that that person gets 90 ETB and is free to return some, all or nothing of that amount to you, or 2. Keep 5 ETB for yourself AND invest 25 ETB in the anonymous person in your own group who receives the tripled amount, 75 ETB and who is free to return some, all or nothing of this amount to you.   If choice 1, go to the **Next experiment**. If choice 2, go **8.a.6** | Code | |  |
| 8.a.6 | What do you prefer?   1. Keep 5 ETB for yourself AND invest 25 ETB in the anonymous person in your own group who receives the tripled amount, 75 ETB and who is free to return some, all or nothing of this amount to you, or 2. Keep 10 ETB for yourself AND invest 20 ETB in the anonymous person in your own group who receives the tripled amount, 60 ETB and who is free to return some, all or nothing of this amount to you.   If choice 1, go to the **Next experiment**. If choice 2, go **8.a.7** | Code | |  |
| 8.a.7 | What do you prefer?   1. Keep 10 ETB for yourself AND invest 20 ETB in the anonymous person in your own group who receives the tripled amount, 60 ETB and who is free to return some, all or nothing of this amount to you, or 2. Keep 15 ETB for yourself AND invest 15 ETB in the anonymous person in your own group who receives the tripled amount, 45 ETB and who is free to return some, all or nothing of this amount to you.   If choice 1, go to the **Next experiment**. If choice 2, go to **8.a.8** | Code | |  |
| 8.a.8 | What do you prefer?   1. Keep 15 ETB for yourself AND invest 15 ETB in the anonymous person in your own group who receives the tripled amount, 45 ETB and who is free to return some, all or nothing of this amount to you. 2. Keep 20 ETB for yourself AND invest 10 ETB in the anonymous person in your own group who receives the tripled amount, 30 ETB and who is free to return some, all or nothing of this amount to you.   If choice 1, go to the **Next experiment**. If choice 2, go to **8.a.9** | Code | |  |
| 8.a.9 | What do you prefer?   1. Keep 25 ETB for yourself AND invest 5 ETB in the trust of the anonymous member of your own group, which we triple to 15 ETB and the anonymous person is free to return some, nothing or all of that 15 ETB to you. 2. Keep 20 ETB for yourself AND invest 10 ETB in the anonymous person in your own group who receives the tripled amount, 30 ETB and who is free to return some, all or nothing of this amount to you.   If choice 1, go to the **Next experiment**. If choice 2, go to **Next experiment**. | Code | |  |
| We will now ask you how you would respond as a receiver of a random envelope from another member in your youth group (amount sent back) and youth group member from another youth group of the same woreda, depending on how big the amount in the envelope you receive is. You know that we have tripled the amount that the other member from your youth group and/or youth group member from another youth group of the same woreda put in the envelope.  **The amounts you decide to return now will be binding for what you have to return when you get the real envelope – but the amount you find there is unknown till you open it as it depends on the decision of the sender (trustor) of that envelope. You will never know who the sender is.** | | | |  |
| How much will you leave in the envelope (return to the sender who is a random **anonymous person in own youth group**) if the amount in the envelope is 90 ETB? | | | ETB |  |
| How much will you leave in the envelope (return to the sender who is a random **anonymous member of another youth group** in the same district (woreda)) if the amount in the envelope is 90 ETB? | | | ETB |  |
| How much will you leave in the envelope (return to the sender who is a random **anonymous person in own youth group**) if the amount in the envelope is 75 ETB? | | | ETB |  |
| How much will you leave in the envelope (return to the sender who is a random **anonymous member of another youth group** in the same district (woreda)) if the amount in the envelope is 75 ETB? **Etc. for smaller amounts 60, 45, 30 and 15 ETB** | | | ETB |  |
