# Supplementary material for: Preferences, trust, and performance in youth business groups
Source: PLoS One. 2021 Sep 20;16(9):e0257637. doi: 10.1371/journal.pone.0257637 (PMC8452030; doi:10.1371/journal.pone.0257637)
Supplement: S2 Appendix — (DOCX) [file pone.0257637.s002.docx]

# Supporting information

## S2 Appendix. Social preference games and single equation models

### Social preference games and categories

Table A. Categorization into social preference categories

| Social preference type | Costless prosocial game (S1 & S2) | Costless envy game (S3 & S4) | | Costly prosocial game (S5 & S6) | Costly envy game (S7 &S8) |
| --- | --- | --- | --- | --- | --- |
| Altruist | 1 | 2 | 1 | | 2 |
| Egalitarian | 1 | 1 | 1 | | 1 |
| Spiteful | 2 | 1 | 2 | | 1 |
| Selfish | 1 or 2 | 1 or 2 | 2 | | 2 |

*Note*: The choices refer to the games in S1 Appendix.

### Single equation models: Social preferences, norm to reciprocate and generalized trust

Table B assesses the extent of correlation between ingroup and outgroup social preferences and individual characteristics, including some parent characteristics. The table shows that the degree of correlation is low for all individual characteristics but gender, age, and education are significant for altruistic and/or selfish group members. We include these three variables as additional controls as a robustness check in the models that relate social preferences, norm of reciprocity, trustworthiness, and trust.

Table C presents results for the relationship between the generalized (outgroup) norm to reciprocate and how it is related to the social preferences of the respondents. We see that altruistic respondents are much more likely to feel extremely obliged to reciprocate than respondents with other social preferences. Respondents with egalitarian preferences also have stronger norms to reciprocate than the others but not as strong as altruists. Model (1) shows that there is substantial explained variation across groups with between-group R-sq. of 0.45 compared to the within-group R-sq. of 0.07.

Table B. Social preferences and individual characteristics

|  | (1) | (2) | (3) | (4) | (5) | (6) | (7) | (8) |
| --- | --- | --- | --- | --- | --- | --- | --- | --- |
|  | Altruistin | Altruistout | Egalitin | Egalitout | Spitefulin | Spitefulout | Selfishin | Selfishout |
| Male, dummy | 0.060*** | 0.035** | 0.025 | 0.032 | 0.005 | 0.022 | -0.109**** | -0.077*** |
|  | (0.021) | (0.014) | (0.021) | (0.020) | (0.009) | (0.019) | (0.024) | (0.025) |
| Age, years | 0.003** | 0.002* | -0.002 | 0.001 | 0.001* | -0.001 | 0.001 | 0.001 |
|  | (0.002) | (0.001) | (0.001) | (0.001) | (0.001) | (0.001) | (0.002) | (0.002) |
| Birth rank | -0.004 | 0.000 | 0.006 | 0.003 | 0.000 | -0.007* | -0.003 | -0.002 |
|  | (0.006) | (0.004) | (0.005) | (0.005) | (0.002) | (0.004) | (0.005) | (0.006) |
| Education, years | 0.006* | 0.002 | 0.000 | 0.002 | 0.002* | 0.005* | -0.007** | -0.008*** |
|  | (0.003) | (0.002) | (0.002) | (0.003) | (0.001) | (0.003) | (0.003) | (0.003) |
| Number of brothers | -0.005 | -0.003 | -0.001 | -0.008 | -0.002 | 0.001 | 0.013 | 0.006 |
|  | (0.007) | (0.005) | (0.007) | (0.006) | (0.003) | (0.006) | (0.008) | (0.007) |
| Number of sisters | -0.005 | -0.002 | 0.003 | 0.000 | -0.002 | 0.005 | 0.000 | -0.008 |
|  | (0.007) | (0.005) | (0.007) | (0.006) | (0.003) | (0.006) | (0.007) | (0.007) |
| Education of parent head | 0.008 | -0.002 | -0.002 | -0.003 | -0.001 | -0.007 | 0.001 | 0.007 |
|  | (0.007) | (0.004) | (0.005) | (0.005) | (0.002) | (0.005) | (0.005) | (0.006) |
| Gender of parent head | -0.030 | -0.028 | -0.016 | 0.029 | 0.000 | -0.017 | 0.013 | 0.028 |
|  | (0.024) | (0.017) | (0.021) | (0.019) | (0.010) | (0.021) | (0.024) | (0.025) |
| Oxen of parent head | 0.0202* | 0.015 | -0.001 | 0.004 | 0.000 | -0.001 | -0.018 | -0.021* |
|  | (0.012) | (0.009) | (0.011) | (0.010) | (0.006) | (0.011) | (0.012) | (0.012) |
| Constant | 0.151* | 0.063 | 0.240**** | 0.054 | -0.002 | 0.202*** | 0.332**** | 0.369**** |
|  | (0.079) | (0.050) | (0.070) | (0.063) | (0.030) | (0.065) | (0.076) | (0.079) |
| N | 2126 | 2126 | 2126 | 2126 | 2126 | 2126 | 2126 | 2126 |
| R-sq | 0.013 | 0.008 | 0.004 | 0.004 | 0.003 | 0.006 | 0.020 | 0.014 |
| adj. R-sq | 0.008 | 0.004 | 0.000 | 0.000 | -0.001 | 0.001 | 0.016 | 0.009 |

*Note:* Linear panel data models with group fixed effects. Standard errors corrected for clustering at the group level. Significance levels: * p<0.05, ** p<0.01, *** p<0.001.

Table C. Social preferences and social norm to reciprocate: Linear panel models with group random effects

|  | (1) | (2) | (3) | (4) |
| --- | --- | --- | --- | --- |
|  | ReciprocOut | ReciprocOut | ReciprocOut | ReciprocOut |
| Outgroup Altruist, Dummy | 0.396*** | 0.338*** | 0.338*** | 0.324*** |
|  | (0.047) | (0.049) | (0.050) | (0.049) |
| Outgroup Egalitarian, Dummy | 0.148** | 0.101* | 0.102* | 0.0960* |
|  | (0.045) | (0.048) | (0.048) | (0.048) |
| Outgroup Spiteful, Dummy | -0.421*** | -0.354*** | -0.359*** | -0.364*** |
|  | (0.051) | (0.053) | (0.054) | (0.053) |
| Outgroup Selfish, Dummy | -0.227*** | -0.159*** | -0.160*** | -0.154*** |
|  | (0.039) | (0.040) | (0.040) | (0.040) |
| Outgroup Altruist share in group |  | 0.522** | 0.361* | 0.348* |
|  |  | (0.188) | (0.170) | (0.173) |
| Outgroup Egalitarian share in group |  | 0.390* | 0.179 | 0.187 |
|  |  | (0.157) | (0.160) | (0.161) |
| Outgroup Spiteful share in group |  | -0.422** | -0.580*** | -0.573*** |
|  |  | (0.160) | (0.152) | (0.151) |
| Outgroup Selfish share in group |  | -0.556*** | -0.400** | -0.392** |
|  |  | (0.130) | (0.130) | (0.129) |
| Male, dummy |  |  |  | 0.109*** |
|  |  |  |  | (0.030) |
| Age, years |  |  |  | 0.002 |
|  |  |  |  | (0.002) |
| Education, years |  |  |  | 0.007 |
|  |  |  |  | (0.004) |
| Tabia Fixed Effects | No | No | Yes | Yes |
| Constant | 2.158*** | 2.271*** | 2.372*** | 2.218*** |
|  | (0.034) | (0.089) | (0.125) | (0.140) |
| N | 2427 | 2427 | 2427 | 2427 |
| R-sq., within | 0.076 | 0.077 | 0.077 | 0.085 |
| R-sq., between | 0.454 | 0.473 | 0.638 | 0.638 |
| R-sq., overall | 0.131 | 0.178 | 0.218 | 0.224 |
| Wald chi2 | 333.7 | 557.3 |  |  |
| P-value | 0.000 | 0.000 |  |  |

*Note*: Dependent variable: Outgroup norm to reciprocate: Obligation to return an amount at least as large as the amount sent by anonymous outgroup member in the trust game: 1=Extremely obliged, 2=Somewhat obliged, 3=Not obliged at all. The model presents marginal effects. Models (3) and (4) include community (*tabia*) fixed effects. The models included youth group random effects and cluster robust standard errors in parentheses. * p<0.05, ** p<0.01, *** p<0.001.

Table C shows that the norm to reciprocate is stronger in groups with more altruists and egalitarians and weaker in groups with more spiteful and selfish members in this outgroup context. This means the outgroup norm to reciprocate is influenced by the composition of the group.

Table D presents the outgroup trustworthiness linear random effects models with the social preference variables and without and with the social norm variables and the group average social preference variables. We see that the explained variation increases substantially when adding the social norm dummy variables while the coefficient on the significant social preference variables are much reduced but remain significant. This indicates that social preferences both have a direct effect and an indirect effect through the norm to reciprocate. By adding the social norm variable the explained within-group variation increases from 0.10 to 0.31 and the explained between-group variation increases from 0.50 to 0.75, showing the importance of the indirect effect and that there is substantial variation in this norm across groups (across-group heterogeneity). For the social preferences group average variables, only the share of spiteful group members is significant indicating that generalized trustworthiness was reduced in groups with more spiteful members. Men were more trustworthy than women and trustworthiness declined with age.

Table E presents linear random effects models for generalized (outgroup) trust while adding controls to the first parsimonious model which includes the social preference variables, outgroup trustworthiness, district fixed effects, and enumerator fixed effects. The added controls include expected return in the trust game, risk tolerance (the invested share in the Gneezy and Potters (1997) risk investment game), the average group social preference variables, and the three individual characteristics. Again the purpose is to assess the explained within-group and between-group variation and how the coefficients of the social preferences and norm variables change when adding the controls.

Table D. Outgroup trustworthiness variation, social preferences and norm to reciprocate

|  | (1) | (2) | (3) | (4) |
| --- | --- | --- | --- | --- |
|  | two1 | two2 | two3 | two4 |
| Outgroup Altruist, Dummy | 0.113*** | 0.0438** | 0.0382* | 0.0393* |
|  | (0.018) | (0.015) | (0.016) | (0.016) |
| Outgroup Egalitarian, Dummy | 0.0583*** | 0.0360** | 0.0398** | 0.0403** |
|  | (0.016) | (0.013) | (0.014) | (0.014) |
| Outgroup Spiteful, Dummy | -0.0901*** | -0.0298* | -0.011 | -0.011 |
|  | (0.014) | (0.012) | (0.013) | (0.013) |
| Outgroup Selfish, Dummy | -0.0639*** | -0.0306** | -0.0237* | -0.0225* |
|  | (0.012) | (0.011) | (0.011) | (0.011) |
| Outgroup Altruist share in group |  |  | 0.037 | 0.043 |
|  |  |  | (0.051) | (0.050) |
| Outgroup Egalitarian share in group |  |  | -0.040 | -0.035 |
|  |  |  | (0.035) | (0.035) |
| Outgroup Spiteful share in group |  |  | -0.128*** | -0.126*** |
|  |  |  | (0.033) | (0.032) |
| Outgroup Selfish share in group |  |  | -0.036 | -0.026 |
|  |  |  | (0.031) | (0.031) |
| No obligation to reciprocate: Base |  |  |  |  |
| Somewhat obliged, dummy |  | 0.125*** | 0.120*** | 0.121*** |
|  |  | (0.008) | (0.008) | (0.009) |
| Strong norm to reciprocate, dummy |  | 0.312*** | 0.304*** | 0.303*** |
|  |  | (0.012) | (0.012) | (0.012) |
| Male, dummy |  |  |  | 0.016* |
|  |  |  |  | (0.008) |
| Age, years |  |  |  | -0.001** |
|  |  |  |  | (0.000) |
| Education, years |  |  |  | 0.000 |
|  |  |  |  | (0.001) |
| Constant | 0.299*** | 0.097*** | 0.131*** | 0.150*** |
|  | (0.019) | (0.018) | (0.025) | (0.028) |
| N | 2427 | 2427 | 2427 | 2427 |
| R-sq., within | 0.101 | 0.313 | 0.313 | 0.314 |
| R-sq., between | 0.496 | 0.746 | 0.757 | 0.766 |
| R-sq., overall | 0.197 | 0.416 | 0.421 | 0.424 |
| Wald chi2 | 763.1 | 2085.6 | 2091.5 | 2221.2 |
| P-value | 0.000 | 0.000 | 0.000 | 0.000 |

*Note*: Dependent variable: Outgroup trustworthiness measured as the share of 30 ETB returned as a trustee in the trust game when the game is played with an anonymous member of another unknown youth group in the same district. The table presents marginal effects from linear panel data models with youth group random effects, district fixed effects and enumerator fixed effects (left out of the table to save space). Cluster-robust standard errors in parentheses, clustering at youth group level. Significance levels: * p<0.05, ** p<0.01, *** p<0.001.

Table E. Outgroup trust, individual member linear models with group random effects and additional controls.

|  | (1) | (2) | (3) | (4) | (5) |
| --- | --- | --- | --- | --- | --- |
|  | trustshareout | trustshareout | trustshareout | trustshareout | trustshareout |
| Altruist, Outgroup, dummy | 0.147*** | 0.130*** | 0.122*** | 0.118*** | 0.116*** |
|  | (0.017) | (0.017) | (0.016) | (0.016) | (0.016) |
| Egalitarian, Outgroup, dummy | 0.038** | 0.036** | 0.029* | 0.034** | 0.033** |
|  | (0.013) | (0.013) | (0.011) | (0.013) | (0.012) |
| Spiteful, Outgroup, dummy | -0.077*** | -0.057*** | -0.029** | -0.016 | -0.018 |
|  | (0.011) | (0.011) | (0.009) | (0.010) | (0.010) |
| Selfish, Outgroup, dummy | -0.038*** | -0.030** | -0.019* | -0.010 | -0.009 |
|  | (0.010) | (0.010) | (0.009) | (0.009) | (0.009) |
| Outgroup Altruist share in group |  |  |  | 0.028 | 0.018 |
|  |  |  |  | (0.040) | (0.040) |
| Outgroup Egalitarian share in group |  |  |  | -0.040 | -0.039 |
|  |  |  |  | (0.033) | (0.033) |
| Outgroup Spiteful share in group |  |  |  | -0.084** | -0.081** |
|  |  |  |  | (0.032) | (0.031) |
| Outgroup Selfish share in group |  |  |  | -0.0670* | -0.067* |
|  |  |  |  | (0.027) | (0.027) |
| Outgroup trustworthiness | 0.367*** | 0.247*** | 0.209*** | 0.202*** | 0.201*** |
|  | (0.025) | (0.027) | (0.024) | (0.024) | (0.024) |
| No obligation to reciprocate: Base | |  |  |  |  |
| Somewhat obliged, dummy |  | 0.074*** | 0.003 | -0.006 | -0.006 |
|  |  | (0.008) | (0.008) | (0.008) | (0.009) |
| Strong norm to reciprocate, dummy |  | 0.144*** | 0.059*** | 0.055*** | 0.050*** |
|  |  | (0.012) | (0.012) | (0.012) | (0.012) |
| Outgroup expected return: <1/3, base | |  |  |  |  |
| One third, dummy |  |  | 0.006 | 0.004 | 0.007 |
|  |  |  | (0.012) | (0.012) | (0.012) |
| Half, dummy |  |  | 0.047*** | 0.046*** | 0.049*** |
|  |  |  | (0.013) | (0.013) | (0.013) |
| More than half, dummy |  |  | 0.0460* | 0.0445* | 0.0464* |
|  |  |  | (0.022) | (0.022) | (0.022) |
| Nothing as I sent nothing, dummy |  |  | -0.182*** | -0.182*** | -0.181*** |
|  |  |  | (0.011) | (0.011) | (0.011) |
| Nothing, although I sent some, dummy |  |  | 0.025 | 0.023 | 0.021 |
|  |  |  | (0.016) | (0.016) | (0.016) |
| Risk tolerance |  |  | 0.038*** | 0.039*** | 0.035*** |
|  |  |  | (0.010) | (0.010) | (0.010) |
| Male, dummy |  |  |  |  | 0.026*** |
|  |  |  |  |  | (0.007) |
| Age, years |  |  |  |  | 0.001* |
|  |  |  |  |  | (0.000) |
| Education, years |  |  |  |  | 0.003** |
|  |  |  |  |  | (0.001) |
| Constant | 0.182*** | 0.117*** | 0.293*** | 0.239*** | 0.185*** |
|  | (0.018) | (0.017) | (0.018) | (0.019) | (0.027) |
| N | 2427 | 2427 | 2427 | 2427 | 2427 |
| R-sq., within | 0.272 | 0.307 | 0.422 | 0.422 | 0.431 |
| R-sq., between | 0.611 | 0.645 | 0.727 | 0.734 | 0.734 |
| R-sq., overall | 0.339 | 0.376 | 0.485 | 0.489 | 0.495 |
| Wald chi2 | 1118.4 | 1683.7 | 3173.6 | 3161.1 | 3143.8 |
| P-value | 0.0000 | 0.0000 | 0.0000 | 0.0000 | 0.0000 |

*Note*: Dependent variable: Outgroup trust, measured as the share invested when playing the trust game with an anonymous group member in another youth group in the same district. The models include district and enumerator fixed effects and youth group random effects. The table presents the marginal effects. Standard errors (in parentheses) are corrected for clustering at the youth group level. Significance levels: * p<0.05, ** p<0.01, *** p<0.001.

Table E shows that we get the expected pattern with shrinking coefficients for social preferences as we add the reciprocity norm, expected returns, and risk tolerance. Outgroup trustworthiness is always highly significantly correlated with ingroup trustworthiness. Those who were more trustworthy towards an unknown youth group member in an unknown group in their own district were also significantly more trusting after having controlled for social and economic preferences and the norm to reciprocate. Highly significant direct effects of altruistic preferences remain showing that those with more altruistic preferences are more trusting. So are also those with egalitarian preferences although to a smaller extent. The presence of more spiteful and selfish members in the groups has detrimental effects on the trusting behavior of all group members. Economic preferences (risk tolerance) and expected returns also matter, indicating that trusting people is perceived to be risky and the amount sent in the trust game is influenced by expected returns. The explained within-group variation increased from 0.31 to 0.42 when adding risk tolerance and expectations on top of social preferences and norm of reciprocity variables, while the between-group explained variation increased from 0.65 to 0.73. This seems to indicate that the between-group variation is particularly large for social preferences and norms. Men were significantly more trusting and age and education were also positively correlated with trust.

Ingroup norm of reciprocity linear panel models are presented in Table F. The results are quite similar to those in the outgroup reciprocity models in Table C. The presence of spiteful members had a particularly strong negative effect on the ingroup norm to reciprocate.

Table F. Ingroup Reciprocity norm models

|  | (1) | (2) | (3) |
| --- | --- | --- | --- |
|  | ReciprocIn | ReciprocIn | ReciprocIn |
| Ingroup Altruist, Dummy | 0.197*** | 0.156*** | 0.0862* |
|  | (0.031) | (0.032) | (0.038) |
| Ingroup Egalitarian, Dummy | 0.128*** | 0.0890* | 0.0829* |
|  | (0.036) | (0.036) | (0.041) |
| Ingroup Spiteful, Dummy | -0.686*** | -0.546*** | -0.476*** |
|  | (0.090) | (0.092) | (0.095) |
| Ingroup Selfish, Dummy | -0.197*** | -0.168*** | -0.170*** |
|  | (0.033) | (0.035) | (0.039) |
| Ingroup Altruist share in group |  | 0.134 | 0.122 |
|  |  | (0.125) | (0.125) |
| Ingroup Egalitarian share in group |  | 0.231* | 0.230* |
|  |  | (0.112) | (0.114) |
| Ingroup Spiteful share in group |  | -1.137*** | -1.085*** |
|  |  | (0.301) | (0.297) |
| Ingroup Selfish share in group |  | -0.218 | -0.199 |
|  |  | (0.127) | (0.128) |
| Outgroup Altruist, Dummy |  |  | 0.139** |
|  |  |  | (0.045) |
| Outgroup Egalitarian, Dummy |  |  | -0.002 |
|  |  |  | (0.040) |
| Outgroup Spiteful, Dummy |  |  | -0.120** |
|  |  |  | (0.044) |
| Outgroup Selfish, Dummy |  |  | -0.024 |
|  |  |  | (0.037) |
| Constant | 2.702*** | 2.705*** | 2.734*** |
|  | (0.051) | (0.092) | (0.091) |
| N | 2427 | 2427 | 2427 |
| R-sq., within | 0.108 | 0.109 | 0.117 |
| R-sq., between | 0.453 | 0.519 | 0.527 |
| R-sq., overall | 0.177 | 0.200 | 0.207 |
| Wald chi2 | 463.1 | 578.1 | 631.5 |
| P-value | 0.000 | 0.000 | 0.000 |

*Note*: Dependent variable: Ingroup norm to reciprocate: Obligation to return an amount at least as large as the amount sent by anonymous ingroup member in the trust game: 1=Extremely obliged, 2=Somewhat obliged, 3=Not obliged at all. The model presents marginal effects. The models included district fixed effects, enumerator fixed effects and youth group random effects. Standard errors are adjusted for clustering at the group level (in parentheses). Significance levels: * p<0.05, ** p<0.01, *** p<0.001.

Table G presents outgroup trustworthiness models. These models demonstrate that the social preference variables primarily work through the norm to reciprocate and that outgroup trustworthiness is a strong predictor of ingroup trustworthiness. Adding the outgroup trustworthiness variable increased the between-group R-square from 0.24 to 0.59 and the within-group R-square from 0.63 to 0.83. Like in the outgroup context men were more trustworthy, and this was even the case after controlling for outgroup trustworthiness. Similarly, age was again negatively associated with trustworthiness.

Table G. Ingroup trustworthiness models: Linear random effects models with additional controls

|  | (1) | (2) | (3) | | | (4) |
| --- | --- | --- | --- | --- | --- | --- |
|  | Twi1 | Twi2 | | Twi3 | Twi4 | |
| Ingroup Altruist, Dummy | 0.0582*** | 0.0462*** | 0.012 | | | 0.009 |
|  | (0.011) | (0.011) | (0.008) | | | (0.008) |
| Ingroup Egalitarian, Dummy | 0.0250* | 0.022 | 0.006 | | | 0.005 |
|  | (0.012) | (0.012) | (0.009) | | | (0.009) |
| Ingroup Spiteful, Dummy | -0.0540* | -0.031 | -0.0438* | | | -0.0397* |
|  | (0.023) | (0.023) | (0.017) | | | (0.017) |
| Ingroup Selfish, Dummy | -0.0374*** | -0.0348** | -0.014 | | | -0.013 |
|  | (0.010) | (0.011) | (0.008) | | | (0.008) |
| Ingroup Altruist share in group |  | 0.101* | 0.033 | | | 0.029 |
|  |  | (0.040) | (0.027) | | | (0.026) |
| Ingroup Egalitarian share in group |  | 0.018 | -0.007 | | | -0.010 |
|  |  | (0.043) | (0.029) | | | (0.028) |
| Ingroup Spiteful share in group |  | -0.230** | -0.089 | | | -0.094 |
|  |  | (0.083) | (0.056) | | | (0.055) |
| Ingroup Selfish share in group |  | -0.007 | -0.005 | | | -0.005 |
|  |  | (0.040) | (0.027) | | | (0.026) |
| No obligation to reciprocate: Base | |  |  | | |  |
| Somewhat obliged, dummy | 0.106*** | 0.103*** | 0.063*** | | | 0.062*** |
|  | (0.015) | (0.015) | (0.011) | | | (0.011) |
| Strong norm to reciprocate, dummy | 0.261*** | 0.253*** | 0.126*** | | | 0.124*** |
|  | (0.015) | (0.015) | (0.012) | | | (0.012) |
| Outgroup trustworthiness, share | |  | 0.648*** | | | 0.633*** |
|  |  |  | (0.014) | | | (0.015) |
| Outgroup trust, share |  |  |  | | | 0.0381* |
|  |  |  |  | | | (0.015) |
| Male, dummy |  |  |  | | | 0.0122* |
|  |  |  |  | | | (0.006) |
| Age, years |  |  |  | | | -0.001*** |
|  |  |  |  | | | (0.000) |
| Education, years |  |  |  | | | -0.001 |
|  |  |  |  | | | (0.001) |
| Constant | 0.151*** | 0.136*** | 0.085*** | | | 0.119*** |
|  | (0.022) | (0.034) | (0.023) | | | (0.026) |
| N | 2427 | 2427 | 2427 | | | 2427 |
| R-sq., within | 0.244 | 0.244 | 0.593 | | | 0.593 |
| R-sq., between | 0.606 | 0.634 | 0.829 | | | 0.840 |
| R-sq., overall | 0.341 | 0.351 | 0.657 | | | 0.660 |
| Wald chi2 | 1094.6 | 1135.9 | 4389.5 | | | 4520.0 |
| P-value | 0.000 | 0.000 | 0.000 | | | 0.000 |

*Note*: Dependent variable: Share returned of 30 ETB if that is the amount received as trustee from an anonymous ingroup member, based on the strategy method. The table presents marginal effects from linear panel data models with youth group random effects, district fixed effects and enumerator fixed effects (left out of the table to save space). Cluster-robust standard errors in parentheses, clustering at youth group level. Significance levels: * p<0.05, ** p<0.01, *** p<0.001.

Finally, the ingroup trust models are presented in Table H. The norm to reciprocate remains highly significant in all models. Outgroup trust was very strongly positively related to ingroup trust and so was ingroup trustworthiness. The social preference variables primarily worked through these variables although altruistic and selfish individual preferences were having significant positive and negative effects, respectively, in all models. None of the group average social preference variables were significant and we left out from the table models with these variables. More risk tolerant members remained significantly more trusting in all model specifications and men were more trusting in the ingroup context, like they were in the outgroup context.

Table H. Ingroup trust, individual member linear models with group random effects and additional controls

|  | (1) | (2) | (3) | (4) | (5) |
| --- | --- | --- | --- | --- | --- |
|  | trustsharein | trustsharein | trustsharein | trustsharein | trustsharein |
| Ingroup Altruist, dummy | 0.108*** | 0.101*** | 0.0275* | 0.0254* | 0.0255* |
|  | (0.013) | (0.013) | (0.012) | (0.011) | (0.011) |
| Ingroup Egalitarian, dummy | 0.038* | 0.033* | 0.009 | 0.008 | 0.009 |
|  | (0.015) | (0.014) | (0.012) | (0.012) | (0.012) |
| Ingroup Spiteful, dummy | -0.112*** | -0.069* | -0.043 | -0.036 | -0.038 |
|  | (0.029) | (0.028) | (0.024) | (0.024) | (0.024) |
| Ingroup Selfish, dummy | -0.050*** | -0.044*** | -0.032** | -0.029** | -0.027* |
|  | (0.013) | (0.013) | (0.011) | (0.011) | (0.011) |
| No obligation to reciprocate: Base | |  |  |  |  |
| Somewhat obliged, dummy | 0.086*** | 0.028 | 0.010 | 0.000 | 0.001 |
|  | (0.019) | (0.020) | (0.017) | (0.017) | (0.017) |
| Strong norm to reciprocate, dummy | 0.225*** | 0.159*** | 0.080*** | 0.060*** | 0.060*** |
|  | (0.019) | (0.020) | (0.018) | (0.018) | (0.018) |
| Ingroup expected return: <1/3, base |  |  |  |  |  |
| One third, dummy |  | -0.039 | -0.019 | -0.021 | -0.020 |
|  |  | (0.029) | (0.025) | (0.025) | (0.025) |
| Half, dummy |  | 0.025 | 0.030 | 0.024 | 0.026 |
|  |  | (0.029) | (0.025) | (0.025) | (0.025) |
| More than half, dummy |  | 0.0614* | 0.0553* | 0.048 | 0.049 |
|  |  | (0.030) | (0.026) | (0.026) | (0.026) |
| Nothing as I sent nothing, dummy |  | -0.223*** | -0.152*** | -0.150*** | -0.150*** |
|  |  | (0.035) | (0.030) | (0.030) | (0.030) |
| Nothing, although I sent some, dummy | | 0.063 | 0.0561* | 0.0568* | 0.055 |
|  |  | (0.033) | (0.029) | (0.028) | (0.028) |
| Risk tolerance |  | 0.0511*** | 0.0298* | 0.0296* | 0.0277* |
|  |  | (0.014) | (0.012) | (0.012) | (0.012) |
| Outgroup trustworthiness, share |  |  | 0.104*** | -0.014 | -0.014 |
|  |  |  | (0.022) | (0.029) | (0.029) |
| Outgroup trust, share |  |  | 0.548*** | 0.541*** | 0.535*** |
|  |  |  | (0.022) | (0.022) | (0.022) |
| Ingroup trustworthiness, share |  |  |  | 0.187*** | 0.185*** |
|  |  |  |  | (0.030) | (0.030) |
| Male, dummy |  |  |  |  | 0.0228** |
|  |  |  |  |  | (0.009) |
| Age, years |  |  |  |  | 0.000 |
|  |  |  |  |  | (0.000) |
| Education, years |  |  |  |  | 0.001 |
|  |  |  |  |  | (0.001) |
| Constant | 0.320*** | 0.349*** | 0.253*** | 0.239*** | 0.219*** |
|  | (0.028) | (0.039) | (0.033) | (0.033) | (0.037) |
| N | 2427 | 2427 | 2427 | 2427 | 2427 |
| R-sq., within | 0.185 | 0.236 | 0.418 | 0.425 | 0.426 |
| R-sq., between | 0.528 | 0.571 | 0.726 | 0.742 | 0.742 |
| R-sq., overall | 0.252 | 0.304 | 0.484 | 0.492 | 0.494 |
| Wald chi2 | 744.4 | 976.7 | 2157.6 | 2264.9 | 2272.1 |
| P-value | 0.000 | 0.000 | 0.000 | 0.000 | 0.000 |

*Note*: Dependent variable: Ingroup trust measured as the share of 30 ETB sent in the trust game when the game is played with an anonymous member of their own youth group. The table presents marginal effects from linear panel data models with youth group random effects, district fixed effects and enumerator fixed effects (left out of the table to save space). Cluster-robust standard errors in parentheses, clustering at youth group level. Significance levels: * p<0.05, ** p<0.01, *** p<0.001.
