# Supplementary material for: Preferences, trust, and performance in youth business groups
Source: PLoS One. 2021 Sep 20;16(9):e0257637. doi: 10.1371/journal.pone.0257637 (PMC8452030; doi:10.1371/journal.pone.0257637)
Supplement: S3 Appendix — (DOCX) [file pone.0257637.s003.docx]

# Supporting information

## S3 Appendix. Group level models: Social preferences, trust and group performance

The following models use aggregate group-level variables to assess the same correlations between-group mean variables as in the earlier group member models. The models in S2 demonstrated large between-group variation that was correlated with included variables. Here we explore this variation further through parametric and non-parametric regressions and graphs. The results confirm many of the same findings as in the earlier models.

Table A. Group average variables used in group-level analysis

| Average group variables | N | Mean | Std. | Min | Max |
| --- | --- | --- | --- | --- | --- |
| Average ingroup trust share | 246 | 0.42 | 0.13 | 0.08 | 0.82 |
| Average outgroup trust share | 246 | 0.23 | 0.10 | 0.00 | 0.53 |
| Average net ingroup trust markup | 246 | 0.19 | 0.08 | 0.02 | 0.58 |
| Average ingroup trustworthiness, share returned | 246 | 0.32 | 0.12 | 0.04 | 0.60 |
| Average outgroup trustworthiness, share returned | 246 | 0.23 | 0.11 | 0.02 | 0.60 |
| Ingroup average Altruist share | 246 | 0.25 | 0.18 | 0.00 | 0.80 |
| Outgroup average Altruist share | 246 | 0.10 | 0.11 | 0.00 | 0.42 |
| Ingroup average Egalitarian share | 246 | 0.18 | 0.14 | 0.00 | 0.63 |
| Outgroup average Egalitarian share | 246 | 0.17 | 0.15 | 0.00 | 0.67 |
| Ingroup average Spiteful share | 246 | 0.03 | 0.06 | 0.00 | 0.33 |
| Outgroup average Spiteful share | 246 | 0.17 | 0.15 | 0.00 | 0.67 |
| Ingroup average Selfish share | 246 | 0.27 | 0.19 | 0.00 | 0.86 |
| Outgroup average Selfish share | 246 | 0.32 | 0.18 | 0.00 | 0.92 |
| Limited obligation to reciprocate, outgroup | 246 | 1.92 | 0.36 | 1.14 | 2.82 |
| Limited obligation to reciprocate, ingroup | 246 | 1.48 | 0.29 | 1.00 | 2.50 |
| Average social relations in group score | 246 | 1.99 | 0.29 | 1.33 | 3.50 |
| Polarized group likelihood score | 246 | 0.08 | 0.11 | 0.00 | 0.75 |
| Average youth group performance score | 246 | 2.41 | 0.46 | 1.44 | 4.20 |
| Average youth group member performance | 246 | 2.39 | 0.39 | 1.56 | 3.88 |

*Source:* 2019 Baseline survey data.

Table B. Average outgroup trust and trustworthiness, social preferences and obligations to reciprocate

|  | (1) | (2) | (3) | (4) | |
| --- | --- | --- | --- | --- | --- |
|  | Average outgroup trust share | Average outgroup trust share | Average outgroup trustworthiness | | Average outgroup trustworthiness |
| Outgroup average Altruist share | 0.301**** | 0.188**** | 0.240**** | 0.055 | |
|  | (0.051) | (0.054) | (0.064) | (0.051) | |
| Outgroup average Egalitarian share | 0.050 | -0.013 | 0.123*** | 0.020 | |
|  | (0.037) | (0.036) | (0.046) | (0.036) | |
| Outgroup average Spiteful share | -0.255**** | -0.142**** | -0.229**** | -0.044 | |
|  | (0.038) | (0.036) | (0.045) | (0.035) | |
| Outgroup average Selfish share | -0.190**** | -0.0899** | -0.226**** | -0.0621* | |
|  | (0.038) | (0.036) | (0.042) | (0.032) | |
| Average obligation to reciprocate |  | 0.139**** |  | 0.228**** | |
|  |  | (0.019) |  | (0.016) | |
| Constant | 0.295**** | -0.025**** | 0.294**** | -0.229**** | |
|  | (0.025) | (0.043) | (0.030) | (0.036) | |
| N | 246 | 246 | 246 | 246 | |
| R-sq. | 0.503 | 0.637 | 0.426 | 0.710 | |

*Note*: Dependent variables: Average outgroup trust share and average outgroup shares returned in the trust game (trustworthiness). Standard errors in parentheses. * p<0.10, ** p<0.05, *** p<0.01, **** p<0.001


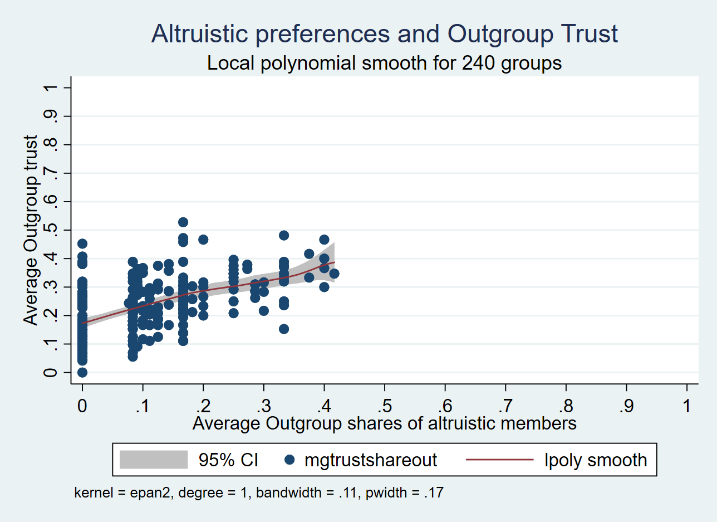

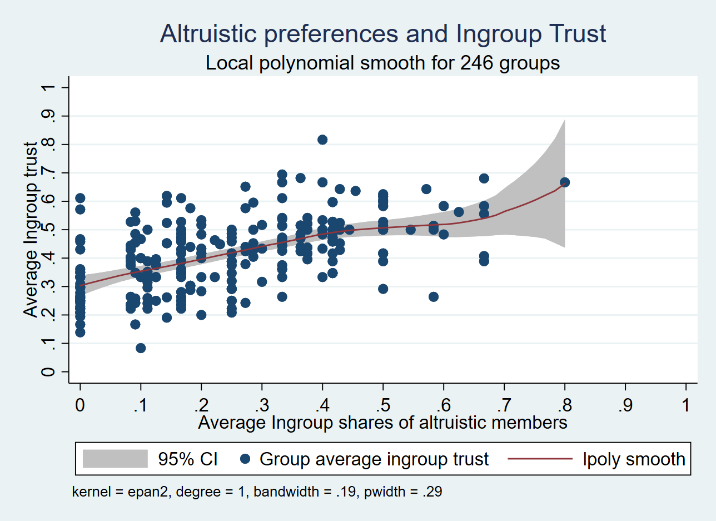


**Fig A**. Group level Altruistic preference distributions and Outgroup and Ingroup Trust


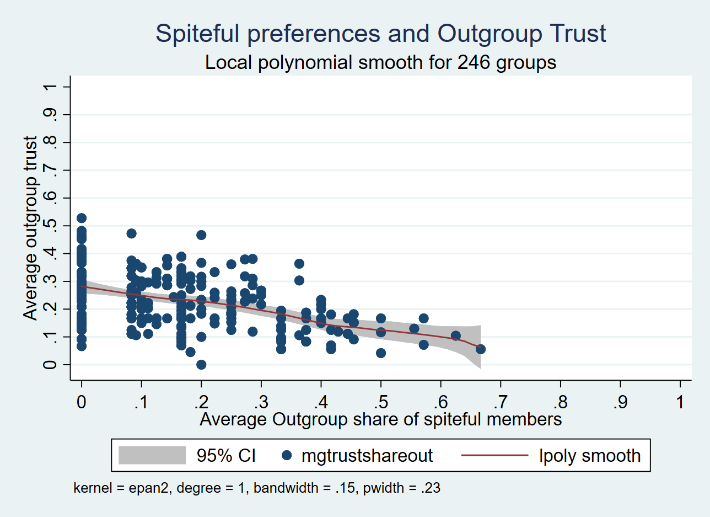

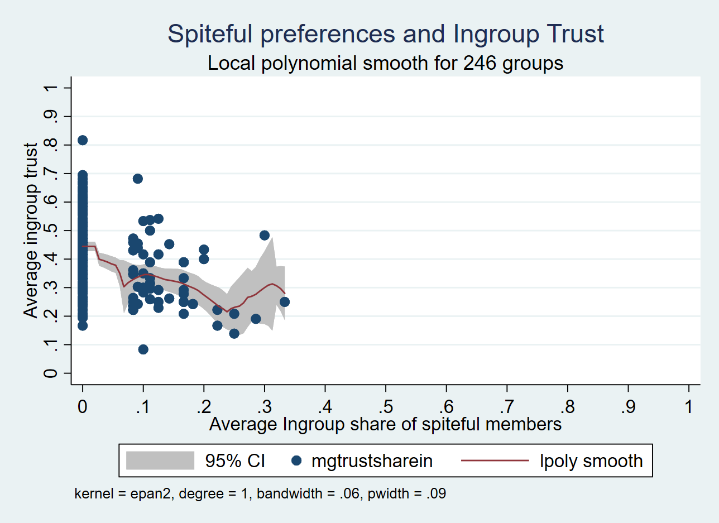


**Fig B**. Group level Spiteful preference distributions and Outgroup and Ingroup Trust


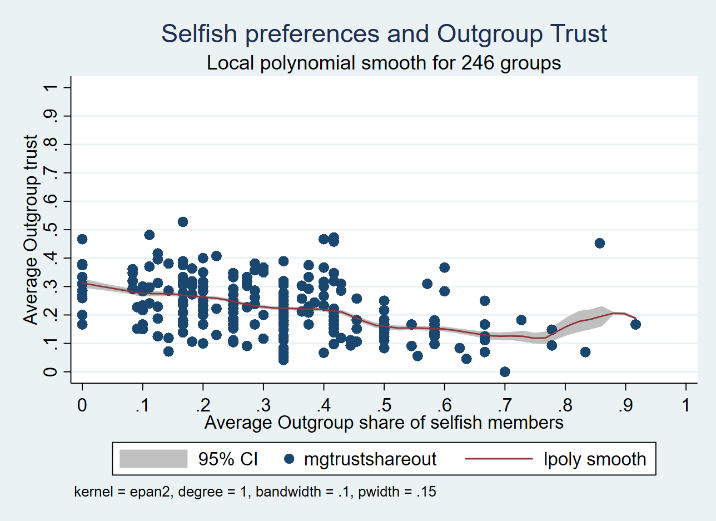

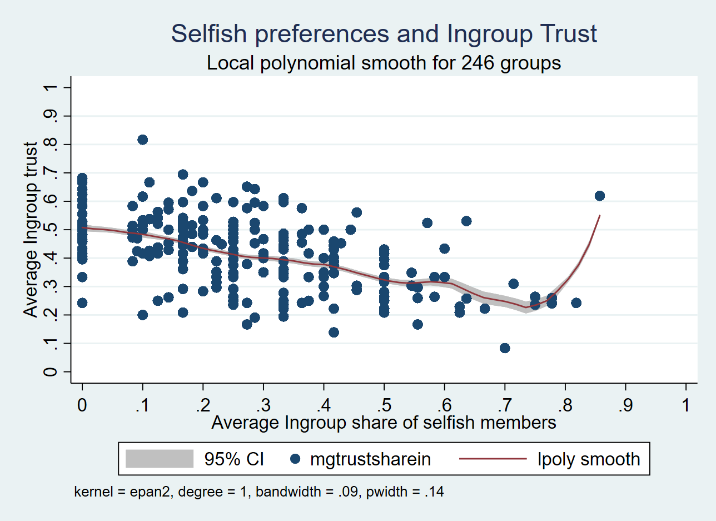


**Fig C**. Group level Selfish preference distributions and Outgroup and Ingroup Trust


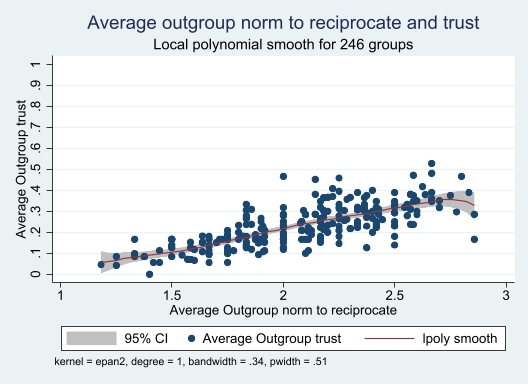

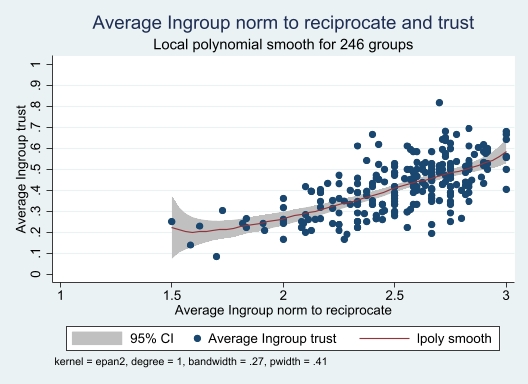


**Fig D**. Average group level Norms to Reciprocate and Outgroup and Ingroup Trust

Table C. Average ingroup trust models

|  | (1) | (2) | (3) | (4) | (5) | (6) |
| --- | --- | --- | --- | --- | --- | --- |
|  | avtrustsharein | avtrustsharein | avtrustsharein | avtrustsharein | avtrustsharein | avtrustsharein |
| Ingroup average Altruist share | 0.265**** | 0.173**** | 0.154*** | 0.062 | 0.062 | 0.062 |
|  | (0.055) | (0.048) | (0.047) | (0.040) | (0.039) | (0.040) |
| Ingroup average Egalitarian share | 0.100* | 0.005 | -0.004 | 0.004 | 0.004 | 0.005 |
|  | (0.052) | (0.046) | (0.046) | (0.039) | (0.038) | (0.038) |
| Outgroup average Spiteful share | -0.534**** | -0.256*** | -0.245*** | -0.185** | -0.176** | -0.190** |
|  | (0.102) | (0.097) | (0.094) | (0.078) | (0.080) | (0.078) |
| Outgroup average Selfish share | -0.154*** | -0.046 | -0.038 | -0.049 | -0.045 | -0.049 |
|  | (0.058) | (0.049) | (0.047) | (0.038) | (0.037) | (0.036) |
| Average obligation to reciprocate |  | 0.180**** | 0.109**** | 0.0423* | 0.0435* | 0.037 |
|  |  | (0.022) | (0.028) | (0.025) | (0.025) | (0.025) |
| Average outgroup trustworthiness |  |  | 0.306**** | 0.166** | 0.156** | 0.156** |
|  |  |  | (0.085) | (0.077) | (0.077) | (0.076) |
| Average outgroup trust |  |  |  | 0.600**** | 0.605**** | 0.610**** |
|  |  |  |  | (0.077) | (0.074) | (0.074) |
| Average group social relations score |  |  |  |  | -0.0285* |  |
|  |  |  |  |  | (0.015) |  |
| Average group polarization score |  |  |  |  |  | -0.108*** |
|  |  |  |  |  |  | (0.039) |
| Constant | 0.390**** | 0.017 | 0.099* | 0.155*** | 0.209**** | 0.173**** |
|  | (0.037) | (0.055) | (0.057) | (0.049) | (0.048) | (0.048) |
| N | 246 | 246 | 246 | 246 | 246 | 246 |
| R-sq. | 0.446 | 0.592 | 0.614 | 0.695 | 0.699 | 0.703 |

*Note*: Dependent variable: Average ingroup trust share by group. Robust standard errors in parentheses. * p<0.10, ** p<0.05, *** p<0.01, **** p<0.001

Table D. Net average ingroup-outgroup trust

|  | (1) | (2) | (3) | (4) | (5) | (6) |
| --- | --- | --- | --- | --- | --- | --- |
|  | netingrtrust | Netingrtrust | netingrtrust | netingrtrust | netingrtrust | netingrtrust |
| Ingroup average Altruist share | 0.014 | 0.006 | 0.001 | 0.062 | 0.062 | 0.062 |
|  | (0.040) | (0.041) | (0.041) | (0.040) | (0.039) | (0.040) |
| Ingroup average Egalitarian share | 0.019 | 0.011 | 0.009 | 0.004 | 0.004 | 0.005 |
|  | (0.038) | (0.040) | (0.040) | (0.039) | (0.038) | (0.038) |
| Ingroup average Spiteful share | -0.170*** | -0.148* | -0.145* | -0.185** | -0.176** | -0.190** |
|  | (0.066) | (0.075) | (0.075) | (0.078) | (0.080) | (0.078) |
| Ingroup average Selfish share | -0.0674* | -0.059 | -0.057 | -0.049 | -0.045 | -0.049 |
|  | (0.036) | (0.039) | (0.039) | (0.038) | (0.037) | (0.036) |
| Average obligation to reciprocate |  | 0.015 | -0.002 | 0.0423* | 0.0435* | 0.037 |
|  |  | (0.018) | (0.025) | (0.025) | (0.025) | (0.025) |
| Average outgroup trustworthiness |  |  | 0.073 | 0.166** | 0.156** | 0.156** |
|  |  |  | (0.077) | (0.077) | (0.077) | (0.076) |
| Average outgroup trust |  |  |  | -0.400**** | -0.395**** | -0.390**** |
|  |  |  |  | (0.077) | (0.074) | (0.074) |
| Average group social relations score |  |  |  |  | -0.0285* |  |
|  |  |  |  |  | (0.015) |  |
| Average group polarization score |  |  |  |  |  | -0.108*** |
|  |  |  |  |  |  | (0.039) |
| Constant | 0.203**** | 0.172**** | 0.192**** | 0.155*** | 0.209**** | 0.173**** |
|  | (0.025) | (0.048) | (0.051) | (0.049) | (0.048) | (0.048) |
| N | 246 | 246 | 246 | 246 | 246 | 246 |
| R-sq | 0.066 | 0.069 | 0.072 | 0.17 | 0.181 | 0.192 |

*Note*: Dependent variable: Average ingroup minus outgroup trust by group. Robust standard errors in parentheses. * p<0.10, ** p<0.05, *** p<0.01, **** p<0.001.
